# The Role of Exosome and the ESCRT Pathway on Enveloped Virus Infection

**DOI:** 10.3390/ijms22169060

**Published:** 2021-08-22

**Authors:** Yichen Ju, Haocheng Bai, Linzhu Ren, Liying Zhang

**Affiliations:** College of Animal Sciences, Key Lab for Zoonoses Research, Ministry of Education, Jilin University, Changchun 130062, China; juyc19@jlu.edu.cn (Y.J.); baihc20@mails.jlu.edu.cn (H.B.)

**Keywords:** exosome, endosomal sorting complex required for transport (ESCRT), enveloped virus

## Abstract

The endosomal sorting complex required for transport (ESCRT) system consists of peripheral membrane protein complexes ESCRT-0, -I, -II, -III VPS4-VTA1, and ALIX homodimer. This system plays an important role in the degradation of non-essential or dangerous plasma membrane proteins, the biogenesis of lysosomes and yeast vacuoles, the budding of most enveloped viruses, and promoting membrane shedding of cytokinesis. Recent results show that exosomes and the ESCRT pathway play important roles in virus infection. This review mainly focuses on the roles of exosomes and the ESCRT pathway in virus assembly, budding, and infection of enveloped viruses. The elaboration of the mechanism of exosomes and the ESCRT pathway in some enveloped viruses provides important implications for the further study of the infection mechanism of other enveloped viruses.

## 1. Introduction

Exosomes are smaller extracellular vesicles (EVs) involved in complex intercellular communication, which were first discovered in sheep reticulocytes [1,2,3]. Exosomes include two subpopulations, large (Exo-L, 90–120 nm) and small (Exo-S, 60–80 nm) exosome vesicles [4]. Exosomes originate from the multivesicular bodies (MVBs) and then are transported to the plasma membrane, fused with the cell membrane, and subsequently released into the extracellular space [3]. They carry various intracellular products, such as nucleic acids and proteins, for information and material exchange between cells [5,6]. Exosomes can incorporate a diverse repertoire of proteins, RNA, and lipids, which may lead to varied biological activity in the recipient cell. Exosome characterization has been supported by the advancements of inclusive databases (e.g., Vesiclepedia, ExoCarta, EVpedia) that amass exosome findings from abundant studies to find distinguishing molecular signatures to specific cell/tissue types [7,8,9]. Consequently, certain proteins, including classic exosomal markers, are present and may be used as exosomes markers following the minimum guidelines set by the International Society of Extracellular Vesicles (ISEV) [10]. An array of exosomes markers such as the tetraspanin proteins (CD9, CD63, and CD81), flotillin-1/-2, ESCRT-related (ALIX and TSG101), RABs, SNAREs, and others have been reported in different disease models [11,12,13,14,15,16,17,18]. However, with a particular focus on budding and infection of viruses, recent studies showed that nucleic acids, proteins, and even virions of enveloped viruses can be wrapped into the exosomes and transmitted between cells by the “free ride” of exosomes.

The endosomal sorting complex required for transport (ESCRT) is several peripheral membrane protein complexes [3,19] that play various roles in cytokinesis, autophagy, retroviral budding, the process of exosome formation and release, and other biological activities [20,21,22,23,24,25,26,27]. In addition, ESCRT-independent mechanisms are also possible for exosome biogenesis [28,29,30]. Whether ESCRT-independent mechanisms play a role remains to be determined. Nevertheless, the cellular ESCRT system is essential for the budding and infection of enveloped viruses. Therefore, it is necessary to further investigate the mechanism of exosomes and the ESCRT pathway in enveloped virus infection.

## 2. Biogenesis of Exosomes and MVBs

**Figure 1:**
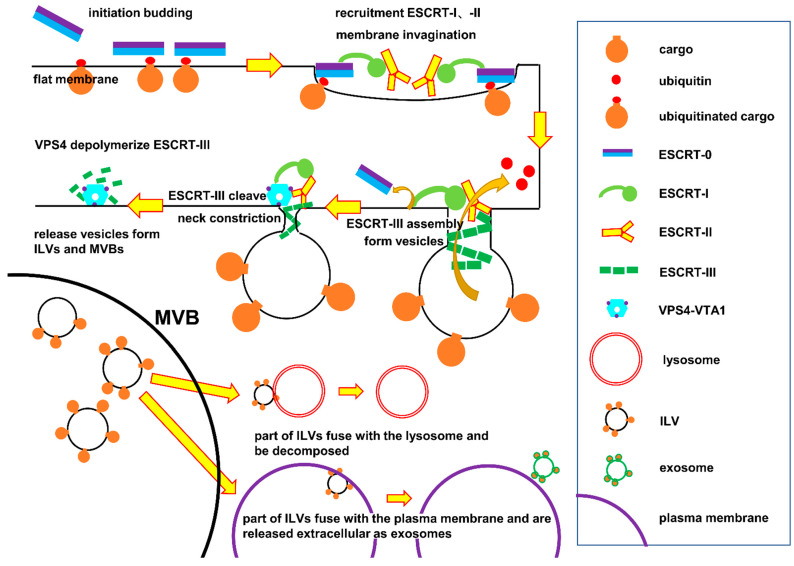
Structure of the ESCRT protein complex and its function in exosome biogenesis [24]. ESCRT-0 initiates the ESCRT pathway by recognizing ubiquitinated cargo and binding to the endosomal membrane. After ESCRT-0 recruits ESCRT-I, ESCRT-I and -II interact with each other and function to invaginate endosome membrane to form initial buds and combine with ubiquitinated cargo to form a cargo-rich area. Then, ESCRT-III is assembly in the neck of the bud. After isolating and classifying ESCRT-I, ESCRT-III drives the budding of vesicles, which are decomposed by the VPS4-VTAL complex.

Exosomes originate from MVBs (Figure 1). The late endosome successively recruits ESCRT-0, -I, -II, -III, and the VPS4 complex. With the help of the ESCRT complex, the endosome membrane invaginates and buds to generate intraluminal vesicles (ILV), and finally forms the MVB [31,32,33,34]. 

The MVB in wild-type yeast is roughly spherical, about 200 nm in diameter, and filled with spherical ILVs, about 24 nm in diameter [35]. In yeast and human cells, when the ESCRT pathway is blocked, it not only interferes with the formation of normal MVB but also shows a unique and abnormal subcellular structure, namely the ‘Class E’ compartments [36,37]. The ‘Class E’ compartments are composed of stacked flat vesicular membranes, which are not connected [35]. Conversely, the presence of the ‘Class E’ compartments can also indicate that the ESCRT pathway is interfered with or blocked. After the formation of MVB, the VPS4–VTA1 complex hydrolyzes ATP to provide energy to depolymerize ESCRT-III for recycling. Some MVBs are degraded after fusion with lysosomes, while the other parts are released into body fluids in the form of small vesicles after fusion with the plasma membrane; these are called exosomes. Studies have shown that the role of ESCRT in MVB biogenesis in eukaryotes is the same as that in yeast.

## 3. Structure and Function of the ESCRT Protein Complex

The ESCRT system consists of ESCRT-0, -I, -II, -III, and vacuolar protein sorting 4–vesicle trafficking 1 (VPS4–VTA1), as well as some accessory proteins such as the ALG-2-interacting protein X (ALIX) homodimer [3,19]. The endosome initiates the ESCRT pathway from ESCRT-0, which is composed of two subunits: hepatocyte growth factor-regulated tyrosine kinase substrate (HRS) and signal transducing adaptor molecule 1/2 (STAM1/2) in eukaryotes (VPS27 and HSE1 in yeast). These subunits interact in a 1:1 ratio via coiled coil GAT (GGAs and Tom) domains [38,39]. Thereafter, these two subunits recognize and bind to the ubiquitinated target, and thus promote its binding to the endosome which is rich in ubiquitinated cargo to be shipped, enabling the endosome to recruit ESCRT-0 to the budding site (Figure 1).

In eukaryotes and yeasts, ESCRT-0 also plays a key role in the recruitment of ESCRT-I to the endosomal membrane, which is crucial for the initiation of MVB-related cargo sorting [40,41]. ESCRT-I was first identified in yeast as a heterotetramer consisting of VPS23, VPS28, VPS37 [42], and MVB12 [43]. Similarly, mammalian ESCRT-I consists of TSG101, VPS28, VPS37, and HMVB12. There are three subtypes of VPS37 (VPS37A, B, and C) and two subtypes of HMVB12 (HMVB12A and B) [44,45]. The length of the ESCRT-I heterotetramer is about 20 nm, in which three subunits are interwoven into a long coil stem with a spherical head formed at one end [46]. Both ends of ESCRT-I interact with ESCRT-0 and ESCRT-II, respectively [47]. Moreover, ESCRT-I also interacts with the ESCRT-II complex.

ESCRT-II is a Y-shaped heterotetramer, with one subunit VPS22 and one subunit VPS36 (EAP30 and EAP45 in mammals) forming the base of Y, each of which binds to one subunit of VPS25 (EAP20 in mammals), which forms the Y arm [48,49,50,51]. In yeast, ESCRT-II interacts with the C-terminal of VPS28 in ESCRT-I through the GLUE (gram-like ubiquitin-binding in EAP45) domain of VPS36 [52,53]. ESCRT-I and ESCRT-II promote the budding of the endosomal membrane to form the initial bud [54,55]. And VPS25 of ESCRT-II binds to VPS20 of ESCRT-III with a high affinity. After VPS20 binds to VPS25, ESCRT-III is activated by ESCRT-II, and components of ESCRT-III begin to recruit and assemble into the endosomal membrane [51], thus activating the shattering function of ESCRT-III. Therefore, ESCRT-II plays a key role in initiating the formation of the ESCRT-III complex.

The main function of ESCRT-III is to aggregate at the neck of the bud formed in the previous step and cleave it, making it enter the endosomal compartment in the form of ILVs to form MVBs [54,55]. Unlike other ESCRT complexes, ESCRT-III does not form stable cytoplasmic complexes, so its crystal structure is currently unclear. ESCRT-III consists of four core subunits: VPS20, SNF7, VPS24, and VPS2 [13]. In mammals, they are known as charged multivesicular body proteins (CHMPs) and include CHMP6, CHMP4A, CHMP4B, CHMP4C, CHMP3, CHMP2A, and CHMP2B. After the ESCRT-III complex aggregates in the neck of the bud to complete its cleaving function, energy is required to depolymerize ESCRT-III to allow it to enter the next cycle. While ATPase associated with various cellular activities (AAA) ATPase VPS4 is responsible for hydrolyzing ATP and providing energy to depolymerize ESCRT-III for recycling [56]. VPS4 is a multimeric mechanoenzyme, that identifies the MIT-interaction Motifs (MIMs) at the C-end of the ESCRT-III subunit through the N-end microtubule interacting and transport (MIT) domain to bind to the ESCRT-III-subunit [56,57]. VTA1 protein is a positive regulator of VPS4, which can promote the polymerization of VPS4 and activate its ATPase activity. When VPS4 performs its biological function in the ESCRT pathway, it is assembled in the form of a polymer which polymerizes into a stable dodecameric containing two hexametric rings and binds with VTA1 protein to form a super complex, namely, the VPS4–VTAL complex [58,59,60]. In conclusion, the ESCRT system plays an irreplaceable key role in the biogenesis of exosomes and MVBs.

## 4. The Role of ESCRT in Enveloped Virus Infection

Enveloped virus infection begins with binding to the plasma membrane of the host cell. Next, the virus enters the host cell for replication and expression, and finally, the new virions leave the host cell at maturity and begin a new cycle of infection [61]. With the discovery that HIV exploits the host cell ESCRT system to assist its budding, numerous studies have shown that a variety of enveloped viruses can hijack the exosomal ESCRT system to assist virus proliferation, budding, and transmission [62,63,64,65,66,67,68]. As a result, the ESCRT system has become an indispensable tool for enveloped virus infection.

### 4.1. The ESCRT System and Enveloped RNA Viruses

At present, the research achievements on the mechanism of enveloped virus budding come mainly from retroviruses. It has been found that retroviruses can release virions from the cell membrane via the ESCRT system [69], and the p6 region of HIV-1 Gag protein (p6^Gag^) is an essential component for the separation and release of new virions from the plasma membrane [70]. Several short sequences were identified in the p6 region that are helpful for the budding of HIV-1 and are known as late assembly (L) domains [70,71,72,73,74]. Mutations in this region would lead to the accumulation of new virions in the inner side of the cell that could not be released. The Gag proteins of retroviruses encode at least three distinct L domains, whose core sequences are PPXY, PT/SAP, and YPX(n)L (where X refers to any amino acid), which recruit different components of ESCRT to form a budding complex for viral release [72,75]. The PT/SAP of the L domain plays a role by combining the TSG101 subunit of ESCRT-I, YPX(n)L works on binding to the ALIX protein, while PPXY binds to members of the ESCRT-related NEDD4 family of E3 ubiquitin ligases [75].

Some enveloped viruses do not depend on ESCRT-II for budding [49,76,77,78], but almost all known viruses that use ESCRT for budding must recruit VPS4, which seems to be the key to viral budding [79]. In addition to retroviruses, A large number of envelope RNA viruses contain L domains, such as *Arenaviruses*, *Rhabdoviruses*, *Filoviruses*, *Reoviruses*, and *Paramyxoviruses* [64]; however, mutations in the viral amino acid sequence of the L domain inhibit the budding and release of the virus. Further studies have shown that the budding and release of these viruses also depend on the host cell ESCRT system [80,81].

#### 4.1.1. The Role of the ESCRT System in the Budding of Retroviruses

The retrovirus budding, especially human immunodeficiency virus type 1 (HIV-1), is particularly similar to the biogenic origin of exosomes [82]. As reported, the TSG101 and ALIX proteins of ESCRT are critical for the budding and release of HIV-1 [83]. Exosome surface marker molecules such as CD63 and CD81 are also involved in HIV-1 budding and infection [84]. HIV-1 RNA and protein can enter the exosome for delivery, and thus retroviruses exploit the exosome formation and release pathways in host cells to produce infectious virions, which was proposed as the “Trojan horse” hypothesis and has been confirmed by subsequent studies, i.e., that HIV-1 hijacks ESCRT-I, -III, and Vps4 to participate in its budding [85]. It was reported that HIV-1 does not require ESCRT-0 and -II for budding [49], presumably because HIV-1’s Gag protein plays a similar role to ESCRT-0 and -PIN. The researchers also used siRNA knockout to demonstrate that budding of HIV-1 in 293T cells does not require ESCRT-II [49]. However, recent studies have shown that efficient production of HIV-1 requires the participation of ESCRT-II [86]. Meng et al. found that during HIV-1 infection, elimination and depletion of ESCRT-II produced distinct effects, as the elimination of ESCRT-II did not eliminate the release of the virus, while depletion of ESCRT-II produced similar effects to depletion of ESCRT-I and -III components, suggesting that ESCRT-II plays an important role in the budding of HIV-1 [86]. Furthermore, ESCRT-I interacts with and activates ESCRT-II through the C-terminal gall of EAP45 and the H0 junction domain, and then recruits CHMP6 of ESCRT-III to form the ESCRT-I-II–CHMP6 complex to play its role [87]. In addition, ESCRT-III is considered to guide membrane remodeling and scission [20,85,88,89,90]. The narrow membrane necks formed during cytokinesis, retroviral, or exosomal budding from membranes [88,89,90]. The scission of these membrane necks from the inner surface is called reverse topology membrane scission, and is directed by the ESCRT complexes, especially ESCRT-III [88,89,90]. 

The HIV-1 Gag protein initiates viral assembly and budding but requires ESCRT-III and VPS4 to separate and release virions from the cell membrane [91]. The Gag protein can promote the formation of buds, which is similar to the role of ESCRT-0 and -II in MVB. Meanwhile, the L domain of the Gag protein containing the PTAP sequence can bind to the UEV domain of ESCRT-I protein TSG101 [92], replacing ESCRT-0 to recruit the ESCRT-I complex. The YPXL module near the C-terminal of HIV-1 Gag protein can interact with the V domain in ALIX [93], thus recruiting ESCRT-I and ALIX in the cell membrane region where the virus is assembled and buds, and then further recruiting ESCRT-III and VPS4 through ESCRT-I and ALIX proteins [94], thus completing the whole process of virus budding.

Different from HIV-1, although equine infectious anemia virus (EIAV) is a vesicular RNA retrovirus, studies have confirmed that TSG101 does not participate in the budding of the virus due to the lack of binding site with EIAV [75,93]. The YPDL sequence of the Gag protein of the virus can recruit ALIX [74] and connect the EIAV to the ESCRT system, which plays a key role in the release of virions at the late stage of EIAV budding [72]. ALIX is involved in the EIAV budding and is responsible for connecting the YPDL of EIAV p9^Gag^ to the host cell ESCRT-III [72]. EIAV infects mammalian cells bud by the interaction of ALIX with EIAV p9^Gag^ and CHMP4 of the host ESCRT-III. The N-terminal Bro1 domain of ALIX binds to CHMP4 and the central V domain binds to the Gag protein [95]. These results suggest that ALIX is essential to the budding of the EIAV.

#### 4.1.2. The IHNV Can Recruit the ESCRT Pathway in Three Ways

Infectious Hematopoietic Necrosis Virus (IHNV) is a kind of *Rhabdovirus*, whose host is fish. IHNV infection poses an enormous threat to salmon farming worldwide because it causes widespread fish die-offs. The L domain of IHNV interacts with multiple host factors to mediate viral assembly and budding, including the ESCRT pathway [96]. The M, G, and L proteins of IHNV contain the L domain, and can directly interact with Nedd4, TSG101, and ALIX in the host ESCRT system [97]. Furthermore, Nedd4, TSG101, and ALIX are all involved in the release of IHNV [97]. However, the three L domains of IHNV are located in different viral proteins, indicating that the absence of one or two viral proteins and the loss of some related factors cannot block the release and infection of IHNV, which may be related to the wide range of hosts of IHNV [98]. In conclusion, PPPH, PSAP, and LXXLF on the IHNV protein can interact with the ESCRT component of fish cells to recruit it to mediate virus budding, but their cell budding sites are not the same [97]. Further research is needed on the biological mechanism of budding virus aggregation in different locations.

#### 4.1.3. The Role of ESCRT in EBOV Budding

Ebola virus (EBOV) is a single-stranded, negative-sense, enveloped RNA virus belonging to the *Filoviridae* family, which can cause hemorrhagic fever syndrome with high mortality. Currently, there is no effective vaccine or treatment for infection and transmission. Recently, it was reported that the ESCRT pathway plays an important role in the budding of EBOV [26,99]. The structural protein VP40 of EBOV plays a central role in the late stage of the assembly and release of the virion. The L domain of VP40 mediates the separation of the virus from the host cell membrane through the recruitment of host ESCRT and its related proteins. VP40 of EBOV contains two overlapping L-domain PPXY sequences and PTAP sequences, similar to the HIV-1 Gag protein, they bind to NEDD4 ubiquitin ligase and TSG101, respectively [100]. Studies have shown that there is a YPX(n)L/I sequence on VP40 of EBOV, which can interact with the ALIX Bro1-V fragment to recruit host ALIX [101]. Moreover, ESCRT-III is critical for the release of Ebola [102]. In recent years, according to the relevant mechanism of ESCRT in EBOV infection, antiviral therapy targeting the interaction between PTAP-TSG101 and PPXY-NEDD4 has been developed [103,104], and it is believed that it will be possible for humans to completely defeat EBOV in the near future.

### 4.2. Exosomal ESCRT Pathway and Enveloped DNA Virus

Apart from enveloped RNA viruses, the ESCRT system also involves the infection of many enveloped DNA viruses, such as insect baculoviruses, hepatitis B, and herpes simplex virus type-1. Some DNA viruses use exosomes to evade cellular immune surveillance, providing an environment for viral proliferation, and thus expanding viral infection.

#### 4.2.1. Insect Baculoviruses’ Invasion and Release from Cells Depends on the ESCRT System 

Baculovirus is an enveloped macromolecule double-link DNA virus with a genome size of about 80–180 kb. In nature, baculovirus uses arthropods as a specific host for transmission and can be used as biological insecticides [105,106], protein expression vectors, and gene transduction vectors of mammalian cells [107,108,109], etc. 

Baculovirions come in two forms: On one hand, Occlon-derived viruses (ODV) are responsible for a viral infection at the individual insect level. ODV is embedded in a proteinaceous occlusion body (OB), and it contains one or more nucleocapsids in an envelope. After oral ingestion of ODV in arthropods, the alkaline environment in their intestines causes the ODV shell to fall off, releasing infectious virions, which initiate infection from insect intestinal epithelial cells. Then the nucleocapsid enters the midgut cells for replication, and the newly formed nucleocapsid buds out of the midgut cells to form the second virion blastomavirus (BV), which invades cells through receptor-mediated endocytosis and mediates systemic infection between cells [106,110]. More than 600 species of baculoviruses have been found, among which *Autographa californica* multiple nucleopolyhedrovirus (ACMNPV) has been extensively studied as a model baculovirus [111]. Early studies have identified several host proteins associated with vesicular transport in ACMNPV budding viruses [112,113]. 

To further determine whether baculovirus infection (invasion and release) is dependent on the host cell ESCRT system, the researchers designed a dominant-negative mutant expressing the VPS4 protein of *Spodoptera frugiperda* cells (Sf9 insect cell) to study its effect on ACMNPV replication. The results confirmed that VPS4 is necessary for both the invasion and release of ACMNPV in insect cells [114]. And recent studies have shown that both the ESCRT-I and ESCRT-III complexes are critical for the effective entry of ACMNPV into insect cells [115]. In addition, several baculovirus cores or conserved proteins (AC11, AC76, AC78, GP41, AC93, AC103, AC142, and AC146) were found to interact with components of VPS4 and ESCRT-III. It is speculated that these viral proteins form an “Egress Complex”, recruiting the components of ESCRT-III to the viral export domain on the nuclear membrane [115]. In conclusion, the host cell ESCRT system is essential for the invasion and release of insect baculovirus infection.

#### 4.2.2. HBV and the ESCRT System

Hepatitis B virus (HBV) is an encapsulated, DNA-containing *Pararetrovirus*, which is one of the most successful pathogens in the world, and there is no radical cure for HBV infection at present [116]. As reported, late endosomes and MVB are used by HBV for assembly and release [117], and the budding and release of HBV virions are related to ESCRT-related molecules [116,118,119,120]. For example, CD63 was found to co-locate with HBV protein in infected liver cells [117]. In addition, all ESCRT-0 components are required for HBV replication, and HRS, the core component of ESCRT-0, plays an important role in HBV transcription [121]. In HBV-producing cells, knockout of TSG101 and VPS28 did not prevent the release of the virus, while knockout of ESCRT-II not only inhibited the production and release of enveloped virions but also impaired the formation of the cell core capsid. Moreover, ESCRT-II was found to co-locate and interact with viral capsid proteins, suggesting that ESCRT-II plays an irreplaceable and important role before HBV buds [122]. In addition, HBV budding on the cell intima and requires the ESCRT-III and VPS4 complex to separate from the membrane [122]. However, the mechanism of how HBV enters the ESCRT pathway is not clear yet. It was reported that Nedd4 ubiquitin ligase interacts with the PPAY sequence of the L domain on HBV capsid efficiently, while γ2-Adaptin, a clathrin adaptor-related protein, establishes the necessary association with both viral capsid and host cell capsule [123,124,125,126], indicating that ESCRT-related proteins involve in the HBV infection. 

#### 4.2.3. Correlation between HSV-1 and the ESCRT System

Herpes simplex virus-1 (HSV-1) is an envelope double-stranded linear DNA virus that uses the host ESCRT to facilitate viral production and transport [127]. The virus can infect humans and a variety of animals, and it is currently highly prevalent in humans [103,104,105].

During infection, viral DNA replication, gene transcription, and nucleocapsid assembly occur in the nucleus. After assembly of progeny virions, they first bud from the nuclear membrane to cytoplasm and obtain a lipid envelope. They then enter the plasma membrane through a second budding and obtain the second envelope before being released into the extracellular environment [128]. HSV-1 exploit nucleoplasmic ESCRTs to promote inner nuclear membrane (INM) remodeling and fission in the first envelopment step [128]. ESCRT-III is recruited to the INM during the budding of HSV-1 from the nucleus, mediating the budding of HSV-1 from the INM and regulating the integrity of the INM [129]. However, the secondary envelope of HSV-1 is dependent on functional VPS4 [130]. Subsequent studies have shown that the expression of any dominant-negative ESCRT-III protein effectively blocked the production of the infectious HSV-1 virus, suggesting that in addition to VPS4 activity, the secondary envelope of HSV-1 is highly dependent on the functional ESCRT-III complex [131]. 

CHMP4 is the most abundant component in the ESCRT-III membrane remolding machine, and it has been found that HSV-1 morphogenesis requires CHMP4C, but not CHMP4A or CHMP4B [132]. In addition, due to several HSV-1 proteins contain the binding motif of ALIX and TSG101, it was suspected that the TSG101 protein of ESCRT-I and the ALIX protein were also involved in the production of infectious HSV-1. However, the results of dominant-negative protein and RNAi detection indicated that ALIX and TSG101 are not required for the secondary envelope of the HSV-1 virus [131]. The specific mechanism by which various proteins of the ESCRT system affect the production of HSV-1 remains to be further studied.

## 5. Conclusions and Perspectives

Exosomes carry cell cargo for cell-to-cell transport, which is also a process that assists in the cell-to-cell transmission of viral nucleic acids and proteins. Envelope viruses can escape the immune system of host cells by encapsulating viral nucleic acids, viral proteins, and even virions into exosomes, regulating the intracellular environment of host cells to help the proliferation of viruses, and using exosomes to follow the circulation of body fluids to various targets of the host body for diffusion and infection. The various components of the host exosome ESCRT system are also involved to varying degrees in the budding and infection of many enveloped viruses, which are hijacked by the virus to assist their proliferation and infection. Therefore, the study of the mechanism of the exosome ESCRT pathway in enveloped virus infection is of great significance for blocking the spread of the virus, inhibiting the proliferation of the virus, and preparing antiviral targeted drugs and virus vaccines. As the mechanism of cystic virus budding and infection via exosome ESCRT continues to unravel, it should soon be possible to target the exosome and ESCRT systems to block and prevent various enveloped virus infections.

## Data Availability

All data generated or analyzed during this study are included in this published article.
